# Asthma spreading and primary diagnostics problems in rural regions of Kazakhstan

**DOI:** 10.1186/2045-7022-5-S2-P22

**Published:** 2015-03-23

**Authors:** Temyrzhan Nurpeissov, Tair Nurpeissov, Gulzada Abdushukurova, Meruert Gazaliyeva, Aizhan Avdugalieva, Alima Shagiyeva

**Affiliations:** 1 SRI of Cardiology and Internal Diseases, Republican Allergological Center, ALMATY, Kazakhstan

## Background

In spite of all efforts, prevalence of bronchial asthma is growing steadily. However, its real level is often much higher than the official statistics.

The aim of this study was to assess the real prevalence of asthma using our innovational algorithm.

## Methods

Screening study amongst the population of two villages of two different rural regions was conducted. 1204 patients became subjects. 681 women and 523 men, their age between 16 and 79 (average 41.3±4.17). During the first stage of the study, they underwent meticulous survey and peak expiratory flow measurement. In the second phase of the study, we examined patients of the study group with the use of an algorithm of asthma management developed by our team. It is a step-by-step strategy, created for general practitioners and medical assistants. This algorithm was designed to facilitate the early detection of asthma.

## Results

According to official statistics, prevalence of asthma in Kazakhstan is 0.3% (3 cases per 1,000 population). However, even the first findings – results of our survey – doubled this figure. Moreover, the implementation of our algorithm showed that real occurrence of asthma is 1.6% (16 cases per 1,000 population). Also, new data acquired suggests that more than half patients with COPD or obstructive bronchitis diagnoses actually has asthma.

## Conclusions

Real prevalence of asthma in Kazakhstan is 5.33 times higher than the official data, many patients was wrongfully diagnosed with COPD or obstructive bronchitis. Even conducting a simple survey, we were able to reveal as much new patients with asthma, as registered. Therefore, the official statistics and diagnostics asthma has clear shortcomings and is in urgent need of improvement.

